# The association of gene polymorphisms with peri-implant mucositis and peri-implantitis: A systematic review and meta-analysis

**DOI:** 10.34172/japid.2025.3432

**Published:** 2025-01-06

**Authors:** Soheil Shahbazi, Saharnaz Esmaeili, Anahita Moscowchi, Reza Amid, Mahdi Kadkhodazadeh

**Affiliations:** ^1^Dentofacial Deformities Research Center, Research Institute for Dental Sciences, Shahid Beheshti University of Medical Sciences, Tehran, Iran; ^2^Research Institute for Dental Sciences, Dental School, Shahid Beheshti University of Medical Sciences, Tehran, Iran; ^3^Iranian Center for Endodontic Research, Research Institute for Dental Sciences, Dental School, Shahid Beheshti University of Medical Sciences, Tehran, Iran; ^4^Dental Research Center, Research Institute for Dental Sciences, Shahid Beheshti University of Medical Sciences, Tehran, Iran

**Keywords:** Alleles, Gene polymorphism, Genetics, Meta-analysis, Peri-implantitis, Systematic review

## Abstract

**Background.:**

The current study aimed to systematically review the existing evidence on potential links between gene polymorphisms and the occurrence of peri-implant mucositis (PIM) or peri-implantitis (PI).

**Methods.:**

The electronic search was executed through six databases in November 2022: PubMed, Embase, Google Scholar, Scopus, Cochrane CENTRAL, and Web of Science. The search sought studies delving into the possible association of gene polymorphisms with PIM or PI. To showcase the effect size, odds ratios along with 95% confidence intervals were used. The meta-analysis was performed on polymorphisms/alleles reported in at least two studies.

**Results.:**

The initial search yielded 2162 results, which were reduced to 1327 following deduplication. After evaluating titles, abstracts, and full texts, 30 studies were deemed suitable for inclusion. Forty-nine gene polymorphisms were examined among 50 PIM patients, 1603 PI patients, and 2407 healthy controls spanning seven ethnicities. The meta-analysis showed that IL-1α -889 (95% CI: 1.070‒2.850, OR=1.746, *P*=0.026), IL-1β+3954 (95% CI: 1.265‒2.851, OR=1.899, *P*=0.002), and OPG -3618 (95% CI: 1.158‒2.983, OR=1.859, *P*=0.010) gene polymorphisms significantly differed between healthy controls and PI patients. However, IL-1β -511, IL-6 -174, OPG -3617, and TNF-α -308 gene polymorphisms did not significantly alter PI risk. Due to insufficient data, performing a meta-analysis on PIM was not feasible.

**Conclusion.:**

The findings suggest that IL-1α -889, IL-1β+3954, and OPG -3618 gene polymorphisms are associated with the predisposition to PI. However, further research among diverse populations is warranted to draw more definitive conclusions.

## Introduction

 Compared to other human organs, teeth are more prone to loss during a person’s lifetime due to multiple reasons, including periodontal diseases, caries, and trauma.^1^ Nevertheless, replacing a missing tooth is no longer a daunting challenge, thanks to the widespread availability of dental implants. From a single missing tooth to complete edentulousness, all can be treated using implants.^2^ In addition to a long-term survival rate exceeding 96% for dental implants,^3^ biological complications may be inevitable in some cases.^4^

 Peri-implant mucositis (PIM) is a reversible state of inflammation that affects the soft tissue near dental implants. If the inflammation is not addressed appropriately, extension toward underlying hard tissues and progressive loss of supporting bone can potentially occur. A different term is assigned to this new state: peri-implantitis (PI).^5,6^ The prevalence of PIM and PI have been reported to range from 19 to 65% and 1 to 47%, respectively.^7^

 As in most periodontal diseases, the activity of bacteria in the biofilm, such as *Fusobacterium* and *Streptococcus* species, stimulates the host immune response.^8^ Multiple proinflammatory mediators and their corresponding receptors participate in the process of inflammation, including interleukins (ILs), tumor necrosis-α (TNF-α), matrix metalloproteinases (MMP), etc.^9^ However, the severity of immune response and the extension of tissue destruction may not be comparable between individuals, even in the presence of similar etiologic factors. A potential explanation for this observed discrepancy could be inter-individual genetic differences.^10^

 Gene polymorphisms are characterized as alterations in the DNA sequence, which must be detectable among at least 1% of a specific population. These alterations, of which single nucleotide polymorphism (SNP) is the most common type, may alter a particular gene’s function or expression.^11,12^ Research has elucidated the association between these genetic alterations and the prevalence of peri-implant diseases (PIDs).^13-23^ For instance, a considerably higher risk of PI has been identified in patients with particular gene polymorphisms of IL-10, IL-1β, osteoprotegerin (OPG), receptor activator of nuclear factor-κB ligand (RANKL), and receptor activator of nuclear factor-κB (RANK).^13-16^ However, existing evidence refutes the associations between the incidence of PI and particular polymorphisms in the DNA sequence of the mentioned proteins.^24-26^

 Accordingly, the relationship between particular gene polymorphisms and their negative impact on the success of dental implants remains unclear.^10,27^ However, if specific sequences of alleles are common among patients with PIDs, clinicians would have the opportunity to assign a more accurate prognosis before implant placement and consider tailored strategies to prevent failures. Therefore, this study aimed to systematically review the current evidence reporting associations between specific genetic polymorphisms and the incidence of PIM or PI.

## Methods

###  Methodology and protocol registration

 The current study was conducted in accordance with PRISMA (preferred reporting items for systematic review and meta-analysis) guidelines,^28^ and the protocol was registered at PROSPERO (International Prospective Register of Systematic Reviews) before initiation (CRD42023367438).

###  Eligibility criteria

 The search strategy was designed in the form of PECO as follows:

- Population (P): Patients who have received dental implants without uncontrolled systemic diseases
- Exposure (E): Presence of polymorphic genotypes
- Comparison (C): Contrasting patients manifesting PI or PIM with healthy controls
- Outcome (O): PI or PIM

###  Inclusion criteria

- Human prospective, observational, and retrospective studies, including cross-sectional, cohort, and case/control studies, randomized or non-randomized clinical trials, and case series
- Investigating patients with PIDs, including PIM and PI
- Reporting the genotype or allele frequencies among diseased and healthy subjects
- English language
- A minimum loading time of six months

###  Exclusion criteria

- Studies that had included patients with uncontrolled systemic conditions such as diabetes mellitus and osteoporosis
- Studies limited to immediate implant placement or loading

###  Search strategy

 The initial search was done through electronic databases of PubMed, Embase, Google Scholar, Scopus, Cochrane CENTRAL, and Web of Science in November 2022. The following search terms were used: (“dental implant” OR “peri-implantitis” OR “periimplantitis” OR “peri-implant disease” OR “periimplant disease” OR “peri-implant mucositis” OR “marginal bone loss”) AND (“polymorphism” OR “variant” OR “mutation” OR “single nucleotide polymorphism” OR “allele” OR “genotype”). The search query was adapted to the guidelines of each database. In addition, a manual search was conducted by scrutinizing the bibliographies of pertinent review articles and journals in the field of dental implants and genetics to retrieve the articles that may have been missed through electronic search. The searched journals were as follows: Journal of Periodontology, Periodontology 2000, Journal of Periodontal Research, Clinical Oral Implants Research, Clinical Implant Dentistry and Related Research, Implant Dentistry, International Journal of Oral and Maxillofacial Implants, Journal of Clinical Periodontology, Journal of Dental Research, Journal of Oral and Maxillofacial Surgery.

###  Screening and data extraction

 The initial results were transferred to EndNote X20 software (Clarivate Company, Philadelphia, USA). The software automatically did the first round of deduplication, and the second round was done manually to omit the machine’s bias. Two independent reviewers (SS and SE) screened the titles and abstracts of the remaining articles while blinded to each other’s decisions. In the next step, the full texts of potentially relevant articles were read comprehensively, and the inclusion/exclusion criteria were applied. Any inter-reviewer disagreement was resolved by consulting a third reviewer (RA). Kappa statistics was used to measure the inter-reviewer agreement throughout title/abstract and full-text screening.

 The subsequent data were gathered from the articles: author and publication year, study design, country, studied polymorphisms, sample size, age and gender, smoking status, history of periodontal disease, plaque index, position of implants, platform type, implant loading time, PI diagnostic criteria, PIM diagnostic criteria, soft tissue biotype, restoration type, DNA sampling site, and main outcome. If any data were missing or not provided in detail, the authors were sent an email and given one month to respond. Any responses received from the authors were documented. In the absence of further clarification from the authors, the study was excluded from the analysis.

###  Quality assessment

 The two independent authors (SS and SE) assessed the quality of each study. “Suggested guidelines for systematic reviews of periodontal genetic association studies”^29^ was implemented for quality assessment. This scoring scale comprises 20 items allocated to five categories: selection, comparability, exposure, study methodology/design, and genetic analyses. A positive answer to each item gains one point, leading to a 0‒20 range for the total quality score. A total score < 6 was deemed as very low quality, 6‒10 was categorized as low quality, 11‒15 was assessed as moderate quality, and 16‒20 was interpreted as high quality. Kappa statistics was used to evaluate the inter-reviewer agreement during the quality assessment.

###  Statistical analyses

 Statistical analyses were carried out to compare alleles and genotypes. The Higgins Index^30^ was used to evaluate the study heterogeneity. According to the Cochrane Handbook, heterogeneity indices falling within the ranges of 0‒40%, 30‒50%, 50‒60%, and 75‒100% were categorized as low, medium, high, and very high levels of heterogeneity, respectively. If no significant heterogeneity (*P* > 0.05) was found, a fixed-effects model (inverse-variance method) was used for the analysis. Otherwise, a random-effects model was applied due to the significant level of the Higgins index (*P* < 0.05). The effect size was presented as odds ratios (ORs) with 95% confidence intervals (CIs). Studies were divided based on the polymorphisms they investigated, and meta-analysis was conducted for polymorphisms/alleles explored in at least two studies. Statistical significance was determined by a P-value of < 0.05. The Egger test^31^ was used to measure the degree of publication bias. The statistical analysis, including forest plot creation, was conducted using Comprehensive Meta-Analysis 2.2.064 software (Biostat Inc., Englewood, NJ).

## Results

###  Study selection

**Figure 1:**
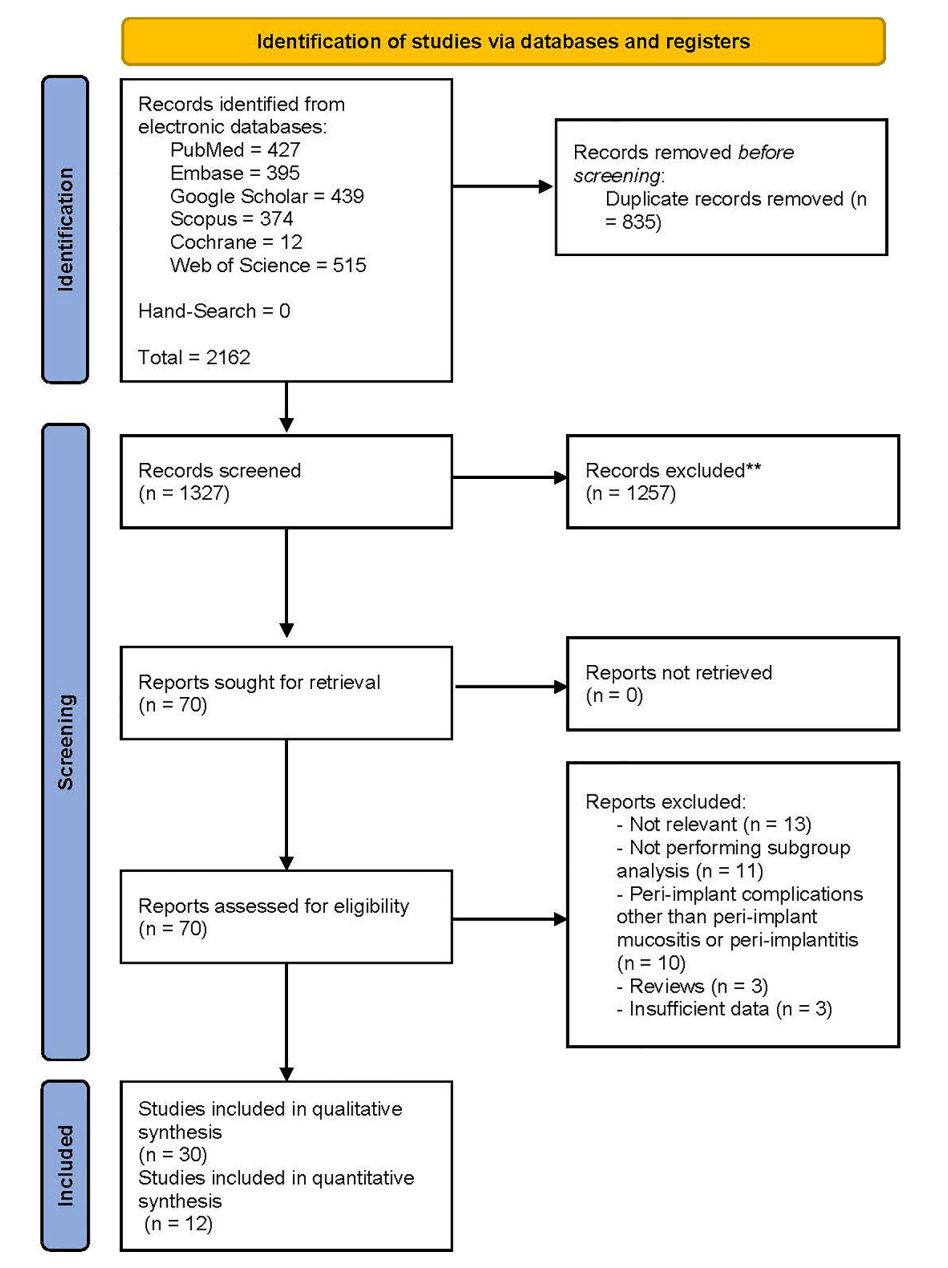
The preferred reporting items for systematic review and meta-analysis (PRISMA) flow diagram

 The PRISMA flow diagram (Figure 1) demonstrates that the preliminary search throughout electronic databases and hand-search yielded 2162 results. The number of records decreased to 1327 following the removal of duplicates. In the next step, title/abstract screening of remaining records left 70 potentially relevant articles. Next, the full texts were scrutinized, and 30 studies were eligible for systematic review. The reasons for excluding 40 records were as follows: irrelevance (n = 13), not performing subgroup analysis (n = 11), investigating peri-implant complications other than PIM or PI (n = 10), reviews (n = 3), and data insufficiency (n = 3). Kappa statistics was calculated at 0.85 for title/abstract and full-text screening, which is considered an almost perfect agreement between reviewers.^32^

###  Study characteristics 

 Comprehensive information about study characteristics and the summary of extracted data are available in Supplementary file (Tables S1 to S3).^13-23,25-26,33-49^ The total number of patients included in the studies was 4060, 50 of which exhibited signs of PIM, 1603 manifested PI, and 2407 were healthy controls. The average age was 51.20 years for patients with PI and 44.41 years for healthy controls. The proportion of females was roughly similar in both groups, with 46.97% in PI-affected subjects and 46.48% in healthy controls. Twenty-six out of 30 studies reported the smoking status of subjects. Regarding the history of periodontal diseases, the number of studies reporting data was 22. The preponderance of research originated from Iran, China, and Brazil, representing case/control and cross-sectional designs. The time that implants were in function before PI or PIM diagnosis varied from 6 to 144 months.

###  Outcome Measures

 Forty-nine gene polymorphisms were investigated in the included studies (Table S1). The most frequently inspected polymorphisms were IL-1B + 3954 and TNF-α -308. As explained before, meta-analysis was performed for gene polymorphisms or alleles scrutinized in at least two studies, delineated in the subsequent paragraphs.

###  Peri-implant mucositis

 Among the 30 reviewed studies, the samples of two studies included 50 PIM patients.^26,43^ The first study investigated the association between RANK (rs3826620), RANKL (rs9594738), and OPG -3618 gene polymorphisms and PIM occurrence, while the other research was focused on IL-6 -174 gene variants. Neither of the studies found significant relationships between the mentioned genetic variations and PIM development. Given the scarcity of studies on each of the aforementioned polymorphisms, executing a meta-analysis was not feasible.

###  IL-1α -889 

**Figure 2:**
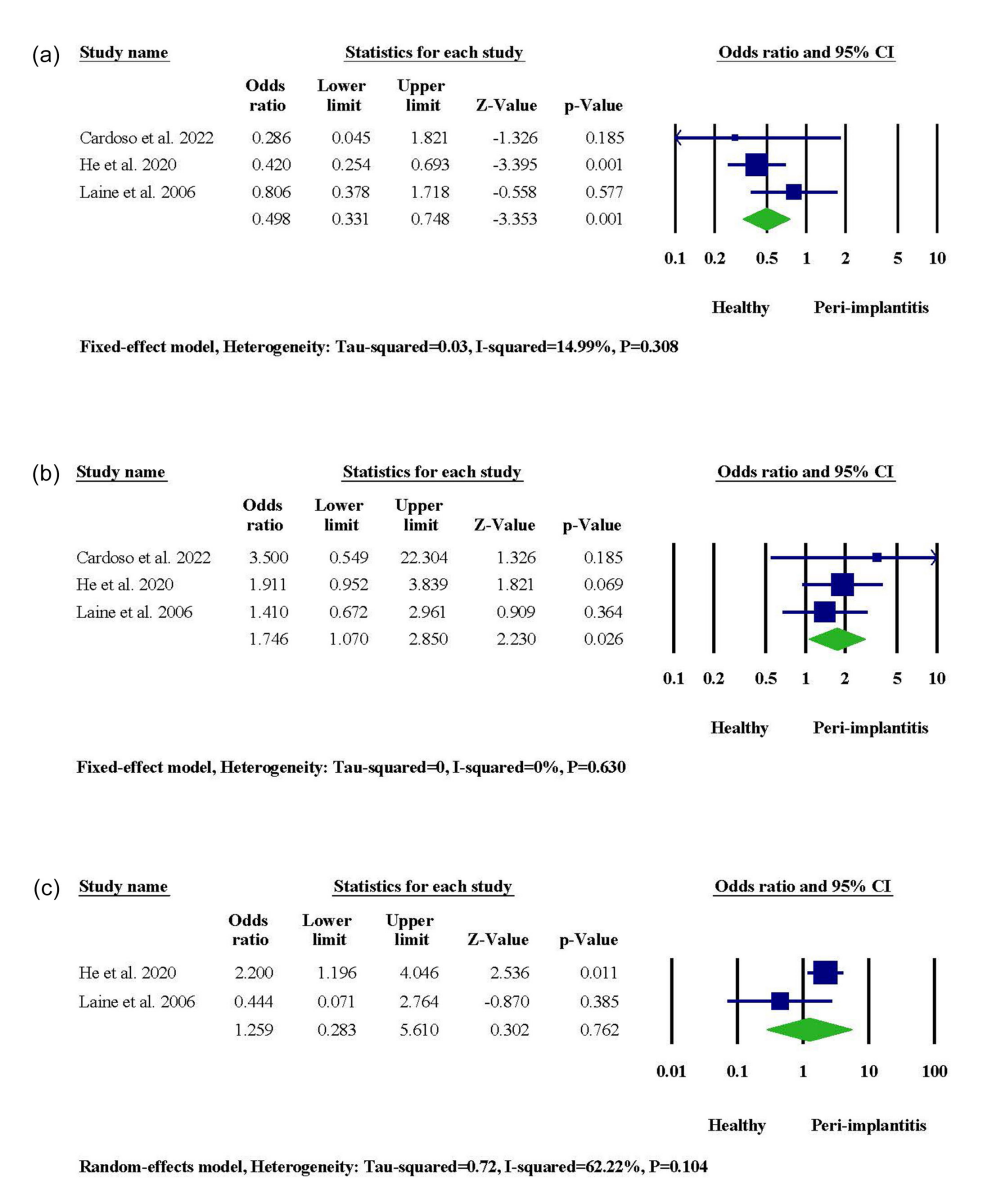
The meta-analysis for the association between IL-1α -889 gene polymorphism and PI risk. (a) CC genotype. (b) CT genotype. (c) TT genotype

 Among 30 studies included in the qualitative synthesis, three investigated the potential association between IL-1α -889 gene polymorphism and the incidence of PI. One of the three studies resulted in a significantly higher risk of PI among non-Han Chinese people with CT and TT genotypes. The meta-analysis showed a significant link between the IL-1α -889 CT genotype and PI risk (95% CI: 1.070‒2.850, OR = 1.746, P = 0.026), while the CC genotype was related to host immunity against the disease (95% CI: 0.331‒0.748, OR = 0.498, *P* = 0.001) (Figure 2). In terms of allele frequency, our results indicated that the presence of the T allele in the genotype of IL-1α -889 was associated with PI incidence (95% CI: 1.398‒2.729, OR = 1.953, *P* = 0.000), and the C allele contributed to peri-implant health (95% CI: 0.303‒0.637, OR = 0.439, *P* = 0.000) (Figure S1).

###  IL-1β + 3954 

**Figure 3:**
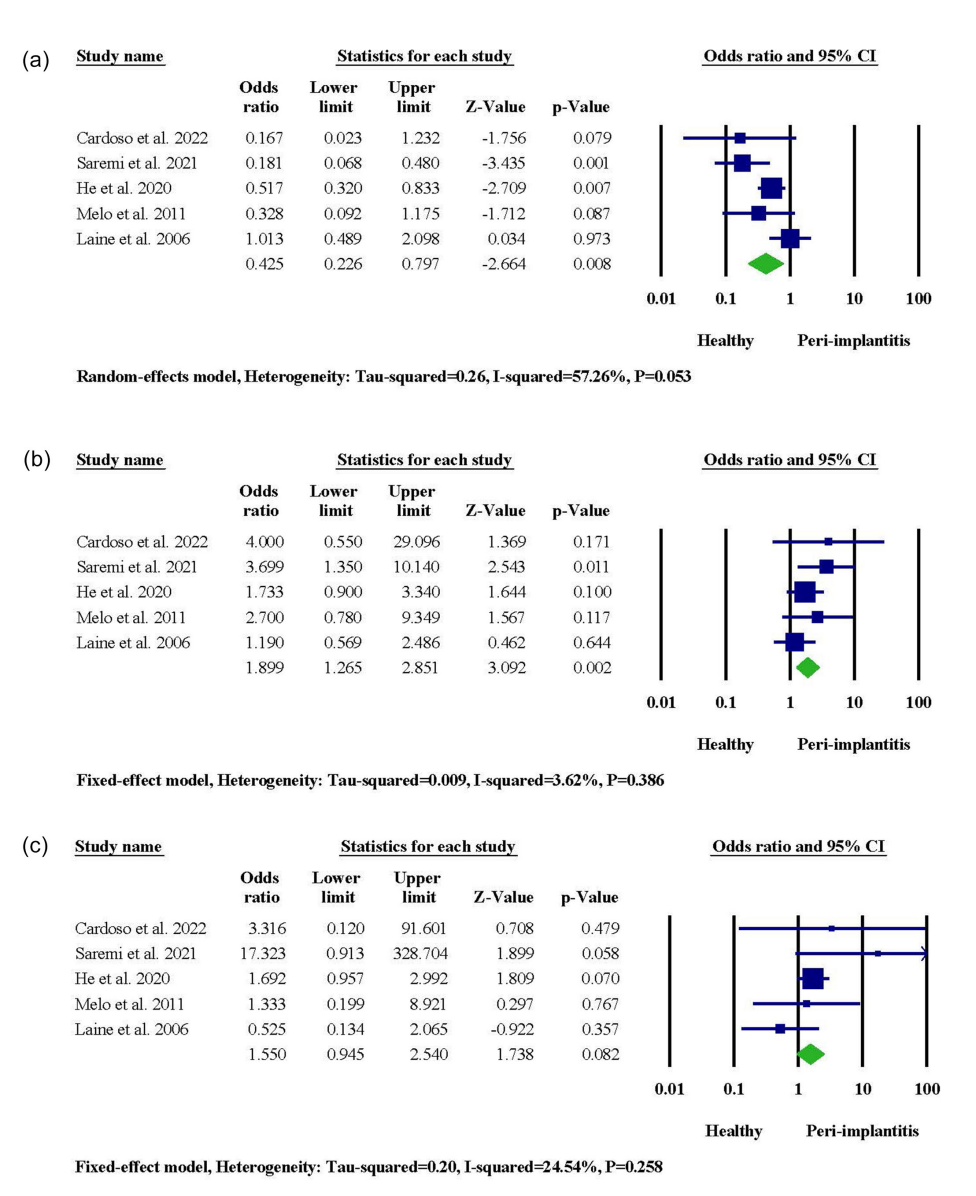
The meta-analysis for the association between IL-1β + 3954 gene polymorphism and PI risk. (a) CC genotype. (b) CT genotype. (c) TT genotype

 A significant association between IL-1β + 3954 gene polymorphism and patients’ susceptibility to PI was reported in two studies. On the contrary, three studies in Portugal, Brazil, and Sweden did not recognize any significant link between genetic exposure and PI risk. When it came to meta-analysis, the IL-1β + 3954 CT genotype was significantly associated with PI occurrence (95% CI: 1.265‒2.851, OR = 1.899, *P* = 0.002), and the CC genotype served as a protective agent (95% CI: 0.226‒0.797, OR = 0.425, *P* = 0.008) (Figure 3). When allele frequencies were compared, it was revealed that the presence of the T allele in the IL-1β + 3954 genotype was linked to an increased risk of PI (95% CI: 1.207‒3.993, OR = 2.195, *P* = 0.010), while the presence of the C allele was associated with healthy condition (95% CI: 0.250‒0.838, OR = 0.457, *P* = 0.011) (Figure S2).

###  OPG -3618

**Figure 4:**
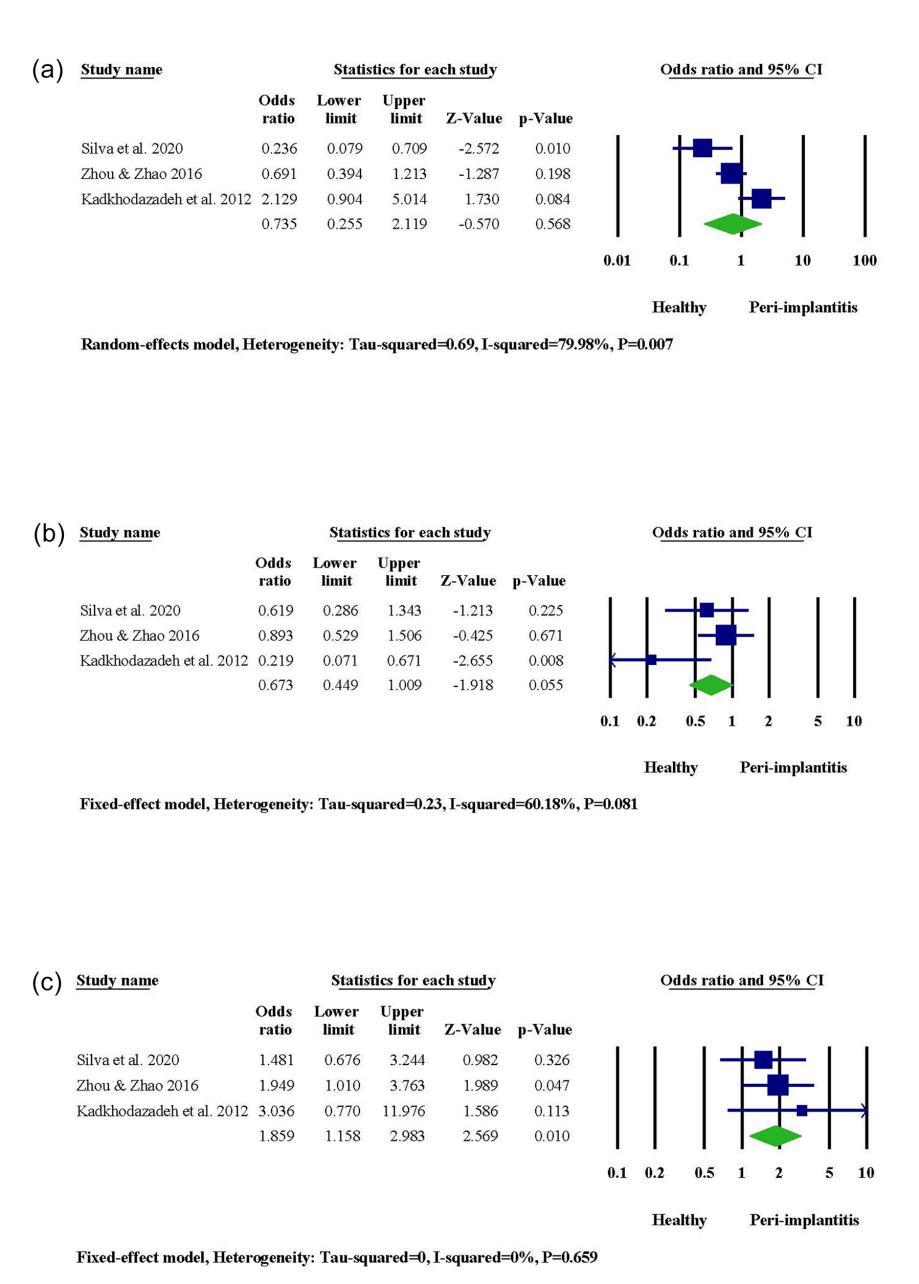
The meta-analysis for the association between OPG -3618 gene polymorphism and PI risk. (a) GG genotype. (b) CG genotype. (c) CC genotype

 According to two out of three studies, PI occurrence was significantly associated with gene polymorphism in the DNA sequence of OPG -3618. However, the statistical significance level was not reached in the other study. Combining results via meta-analysis represented augmented PI susceptibility in the presence of CC genotype (95% CI: 1.158‒2.983, OR = 1.859, *P* = 0.010) (Figure 4). A higher risk of PI was observed in subjects carrying the C allele (95% CI: 1.090‒2.019, OR = 1.483, *P* = 0.012). In contrast, the G allele contributed to a decreased risk of PI (95% CI: 0.495‒0.918, OR = 0.674, *P* = 0.012) (Figure S3).

###  IL-1β -511

 Polymorphic genotypes of IL-1β -511 were not related to the prevalence of PI, as stated in two studies conducted in Brazil and Sweden.^46,49^ Consistently, the meta-analysis did not discover any particular association between polymorphic genotypes/alleles and PI incidence (Figure S4).

###  IL-6 -174

 As reported in two studies, the frequency of polymorphic genotypes of IL-6 -174 was differential between PI-suffering patients and healthy controls.^38,43^ In contrast, one study refuted any potential link between polymorphic genotypes and disease development.^46^ The meta-analysis revealed that polymorphic genotypes/alleles do not contribute to a healthy or diseased peri-implant condition (Figure S5).

###  OPG -3617

 The results of two studies were in favor of an insignificant association between OPG -3617 genetic polymorphisms and the incidence of PI.^16,39^ The current meta-analysis supported the assertion that variations in the DNA sequence of OPG -3617 are not related to PI development (Figure S6).

###  TNF-α -308

 One out of five studies endorsed a significant association between polymorphisms in the genotype of TNF-α -308 and the risk of PI among a Serbian population.^38^ The other four studies did not identify the mentioned association between genetic variations and disease occurrence.^13,36,47,48^ The meta-analysis in the current study also revealed an insignificant relationship between genetic variants and PI occurrence (Figure S7).

###  Quality assessment 

 As demonstrated in Table S4, the minimum total quality score was 6, while the maximum was 13. Based on the total scores, 25 studies exhibited moderate quality, while 5 were low-quality. The Kappa statistics was calculated at 0.90 for quality assessment, deemed an almost perfect agreement between reviewers.^32^

## Discussion

 PIM and PI are two common complications affecting dental implants. The former is confined to soft tissues, while the latter is the extended state, which involves both soft and hard tissues.^5,6^ Gene polymorphisms, defined as alterations in the genotypes present in a considerable portion of a population, are associated with susceptibility to PIM and PI in numerous studies.^11-16^ As a consequence, the current study aimed to systematically review the existing evidence on any potential association between genetic variations and the incidence of PIM and PI.

 Among 30 included studies, the relationship between 49 distinct gene polymorphisms and the incidence of PIM and PI was evaluated. The meta-analysis revealed that polymorphisms in the DNA sequence of IL-1α -889, IL-1β + 3954, and OPG -3618 may cause significant differences in patients’ susceptibility to PI. On the contrary, patients carrying polymorphic genotypes of IL-1β -511, IL-6 -174, OPG -3617, and TNF-α -308 may not experience a significantly higher or lower risk of PI.

 Delving deeply into the pathogenesis and the role of immune responses in the development of PIM and PI is crucial for understanding the findings of this study. The immune response to bacterial challenge is more aggressive and rapid in the soft tissue surrounding implants compared to teeth. During the first weeks, the host’s response to bacterial challenges is similar in peri-implant mucosa and gingiva. However, if bacterial accumulation progresses for months, the apical extension and magnitude of inflammatory infiltration will be more pronounced in peri-implant mucosa.^50^ Variations in the inflammatory and immunological responses to bacterial infections can impact an individual’s susceptibility to PIDs. The sequence of inflammatory mediators released by the host in reaction to such infections may lead to the destruction of connective tissue and bone, a process influenced by genetic determinants.^51^ Identifying genetic factors and their ability to modulate the intensity of the host response could be instrumental in both treating and preventing PIDs.

 The results of the current meta-analysis indicated that the T allele and CT genotypes of IL-1α -889 and IL-1β + 3954 might be associated with a higher chance of PI development. The IL-1α -889 and IL-1β + 3954 genes are responsible for regulating IL-1α and IL-1β, respectively. These two genes are adjacent on the q arm of chromosome 2, binding with the same receptor of IL-1R1.^52,53^ Upregulation of IL-1α and IL-1β plays a key role in the development of periodontal inflammation through triggering cell chemotaxis, collagen destruction, and bone resorption.^53^ The presence of the T allele in the DNA sequence of IL-1α -889 and IL-1β + 3954 is significantly associated with early implant failure.^54^ According to a systematic review by Mohammadi et al,^55^ composite genotypes of IL-1α -889/IL-1β + 3953 were linked to the risk of PID. The same association was identified for IL-1β + 3954 gene polymorphism, particularly the CT genotype. However, some studies assert that evaluating the mentioned genetic variations may not necessarily forecast a patient’s susceptibility to peri-implant or periodontal diseases.^42,56,57^

 Our meta-analysis refuted any significance regarding the association between IL-1β -511 and IL-6 -174 gene polymorphisms and patients’ susceptibility to PI. Both osteoblasts and osteoclasts produce IL-6 in response to the activity of local bone-resorbing substances. Accordingly, it potentially induces bone resorption alone or in conjunction with other osteoclastic agents.^58,59^ Although peri-implant crevicular fluid (PICF) levels of IL-1B and IL-6 rise during PID,^60^ the correlation between the concentration of these two mediators in PICF and experiencing a diseased or healthy condition was rebutted by Melo et al.^46^ In the same study, the influence of IL-1β -511 and IL-6 -174 gene polymorphisms on the incidence of PIDs was found to be insignificant.^46^ Consistently, IL-6 -174 genetic variations were neither associated with PID development nor early implant failure.^43,61^ In addition, Laine et al^49^ did not identify IL-1β -511 gene polymorphism as a significant risk factor for PI. The studies included in the meta-analysis of IL-1β -511 gene polymorphism were conducted on Brazilians and Caucasians in Sweden. Moreover, IL-6 -174 gene polymorphism was evaluated among Serbians and Brazilians. Consequently, further research is required on various populations to achieve outcomes of high statistical reliability.

 The present meta-analysis showed a significant association between the C allele and CC genotype of OPG -3618 and PI risk. However, the same relationship was not recognized between OPG -3617 gene polymorphism and the occurrence of the disease. Exhibiting structural homology to RANK, OPG can block the RANK receptor, i.e., RANKL, to halt the cascade of events leading to osteoclast differentiation and bone resorption.^62^ The imbalance in the RANKL/OPG ratio has been recorded in patients suffering from PIDs, confirming the role of these two mediators during osteo-immunoinflammatory response contributing to PI.^60^ As a consequence, OPG has been identified as a potential candidate for periodontal diagnosis.^63,64^ In line with our findings, Zhou and Zhao^39^ reported a significant association between OPG -3618 gene polymorphism and the risk for PI, while the association was insignificant for OPG -3617. In an Iranian population, a significant connection between the polymorphic genotype of OPG -3618 and the presence of PI was also found.^16^ In contrast, E Silva et al^26^ did not realize any significant association between OPG -3618 genetic variations and the incidence of PI or PIM in a Brazilian population. Among the included studies, OPG -3617 genotyping was performed in two studies on the Chinese Han population and Iranians. Accordingly, there is a severe shortage of studies on the mentioned populations to achieve conclusive results.

 The meta-analysis revealed no significant association between SNP in the DNA sequence of TNF-α -308 and the occurrence of PI. TNF stimulates various events, including chemokine expression, inflammatory mediator production, osteoclastic activity, and MMP release. The mentioned mechanisms contribute to inflammation, bone loss, connective tissue destruction, and impaired periodontal repair.^65^ Salivary levels of TNF-α elevate in patients with periodontitis, making this biomarker a promising tool for screening and diagnosing periodontal diseases.^66^ In line with this, Ghassib et al^67^ introduced TNF-α as an adjunct to differentiating healthy periodontium from PIM- and PI-affected sites. Regarding genetics, Jamshidy et al^24^ concluded that TNF-α -308 gene polymorphism significantly increased the risk of PID among Asians. In another systematic review, TNF-α genotyping is highlighted as a prognostic marker for implant treatment.^68^ Regarding implant failure, the evidence on TNF-α genotyping did not support the association between polymorphisms and this consequence.^27,69^ Three of the included studies investigated TNF-α -308 genetic variations. However, they were performed on three continents: the Iranian, Chinese, Serbian, and Brazilian populations. The diversity of samples in the studies may contribute to insignificant pooled meta-analysis results.

 Despite studies eligible for meta-analysis, some studies reported statistically significant results after investigating various genotypes and their link to PIDs. Chang et al^33^ highlighted the possible link between a particular polymorphic genotype of epidermal growth factor (EGF) and higher protection against PI. The defensive role of the G allele was confirmed in two other studies investigating generalized aggressive periodontitis.^70,71^ Additionally, the presence of the T allele in the sequence of fibroblast growth factor 3 (FGF3) (rs4631909) was shown to be significantly associated with a healthy peri-implant condition.^40^

 Regarding the IL-16 (rs4072111) gene, a significant association between genetic variations and PI risk was identified among the Chinese Han population.^34^ However, the relationship between IL-16 (rs4072111) genetic variants and periodontitis susceptibility was insignificant in another study on Brazilians.^72^ Moreover, particular gene polymorphisms of IL-10 -819 and IL-10 -592 potentially contribute to PI pathogenesis among Iranians.^13^ In contrast, Jamshidy et al^24^ denied any significant association between PID and the mentioned polymorphisms in Asian subjects. It should be noted that ethnicity has been identified as an influential factor in the risk of periodontitis. In detail, although the IL-10 -592 was significantly linked to periodontitis risk in the overall population, IL-10 -819 genetic variations were associated with periodontitis only in specific ethnicities.^73^ According to Petkovic-Curcin et al,^38^ the prevalence of IL-10 -1082 polymorphic genotypes was different between PI patients and healthy controls. However, the mentioned significant difference was not identified in a systematic review comparing PID risk in patients with or without gene polymorphism IL-10 -1082.^24^ Regarding periodontitis, a significant association with IL-10 -1082 gene polymorphism was reported only for Caucasians.^73^ Kadkhodazadeh et al^44^ have highlighted the potential contribution of IL17 (rs10484879) CC genotype to the pathogenesis of PI and periodontitis.

 Qi et al^35^ introduced CXCR2 (rs2230054) gene polymorphism as a risk factor for PI occurrence, while in another study, there was no significant association between SNPs in the CXCR2 genotype and chronic periodontitis incidence.^74^

 The association between chronic periodontitis and PI risk with Fc-gamma receptor IIa (Fc-FCGRIIa) (rs1801274), FCGRIIIa (rs396991), and FCGRIIIb (rs1050501) gene polymorphisms is significant among Iranians.^37^ On the contrary, the association of FCGR genetic variations with PI or periodontitis did not appear significant in other studies.^75,76^ In addition, Caucasians carrying polymorphic genotypes of FCGRIIa (rs1801274) and FCGRIIIa (rs396991) represented higher susceptibility to periodontitis, while gene polymorphisms of FCGRIIIb (rs1050501) may give rise to aggressive periodontitis.^77^

 The presence of the T allele in the DNA sequence of CD14 -159 (rs2569190) has been recognized as a defensive factor among Serbians.^38^ Consistently, Rakic et al^78^ calculated a fivefold increased risk of PI among Caucasians carrying CC genotype. While evidence supports the potential role of CD14 -159 gene polymorphism in the development of periodontitis,^79,80^ few studies have refuted the involvement of this genetic variation in periodontitis incidence.^81,82^

 MiR146a (rs2910146) and MiR499 (rs3746444) gene polymorphisms have been introduced as possible genetic determinants for chronic periodontitis and PI occurrence.^45^ Although MiR146a gene polymorphism was not significantly associated with chronic periodontitis, its specific haplotype combinations with MiR196a were inversely linked to chronic periodontitis.^83^ Investigating an Indian population, Venugopal et al^84^ concluded that polymorphic genotypes of MiR499 were related to a higher risk of chronic periodontitis.

 As stated by Coelho et al,^40^ the TT polymorphic genotype of BMP4 (rs2761884) decreased the risk of PI significantly. Furthermore, another study has suggested the impact of BMP4 genetic polymorphism on early marginal bone loss surrounding dental implants.^85^ It has been reported that particular polymorphic genotypes of RANKL (rs9533156) can give rise to PI development among Iranians.^14^ However, SNPs in the genotype of RANKL exhibited no significant association with aggressive periodontitis in a Japanese population.^86^ The link between BRINP3 (rs1342913) and PI susceptibility has been delineated by Casado et al.^41^

 Among the 30 included studies, two scrutinized the possible association between gene polymorphisms and the incidence of PIM.^26,43^ E Silva et al^26^ assessed RANK (rs3826620), RANKL (rs9594738), and OPG -3618 genetic variations among PIM-affected patients. They reported that these polymorphisms were not significantly associated with PIM risk. In the other study, Casado et al^43^ found no significant relationship between polymorphic genotypes of IL-6 -174 and a higher or lower rate of PIM occurrence. PIDs are considered counterparts of gingivitis and periodontitis occurring around dental implants. Consequently, the pathogenesis of these diseases exhibits high degrees of similarity.^87^ Given this, any significant association between IL-6 -174 gene polymorphism and the risk for periodontitis and gingivitis was refuted by Salman et al.^88^ Moreover, the link between SNPs in RANK, RANKL, and OPG genes and aggressive periodontitis was rebutted in the Japanese population.^86^

 Although gene polymorphisms have been introduced as potential risk factors for PIDs, other factors may contribute to disease progression. One of these factors is the smoking status of patients. It has been shown that the risk of marginal bone loss rises as the daily smoking of patients increases. In detail, smokers, whether less or more than 10 cigarettes a day, exhibited higher levels of marginal bone loss than non-smokers.^89^ Furthermore, the risk of failure is 140.2% higher in smokers receiving dental implants compared to non-smokers.^90^ The other important factor is the history of periodontitis. While the implant survival rate might not exhibit substantial divergence between individuals with a history of periodontitis and those without, the former group may experience higher marginal bone loss and PI incidence.^91^ Consistently, Sgolastra et al^92^ introduced periodontitis as a remarkable risk factor for implant loss and PI. Another study investigated implant surface characteristics as a possible cause of peri-implant bone loss. The results indicated that the peri-implant bone loss was smaller around less rough fixtures.^93^ However, in a randomized clinical trial comparing two implant systems, marginal bone loss was significantly higher around machined-surface implants than rough-surfaced ones.^94^ The last factor associated with PID progression is a clinician’s experience. The surgeon’s experience regarding the number of implants placed might significantly influence the implant failure rate.^95^ The experience‒failure relationship was also endorsed in other studies.^96,97^ Other potential variables that might affect the progression of PID are the implant loading time, the choice between cement- or screw-retained prostheses, and the decision for either tissue- or bone-level placement of the implant.^98-100^ In brief, further comprehensive studies are needed to take the mentioned factors into account simultaneously with genetic predisposition to achieve more conclusive results with higher accuracy.

 The periodontitis treatment can potentially follow two distinct paths: remaining in a remission phase or exacerbating into an active phase of periodontal destruction.^101^ The remission phase is characterized by a relative reduction in inflammation and a slight improvement in attachment levels following treatment.^102^ A parallel classification applies to the phases of PI. It has been established that there are notable differences between remission and active PI, including variations in microbiota and host response. Specifically, *Porphyomonas*, *Fusobacterium*, *Treponema*, and *Tannerella* dominate the microbial population during the active destruction phase, whereas lactic acid bacteria prevail in PI sites during remission.^103^ Given that different cytokines are active during various stages of periodontal diseases, the expression levels of these cytokines can markedly fluctuate between each stage.^104^ In the case of PI, inflammatory infiltration is pronounced and primarily involves plasma cells.^105^ Accordingly, each gene polymorphism may play a vital role in a particular stage of the disease, which implies that the stage of the disease should be considered when researchers are recruiting samples for their studies.

 It is crucial to elaborate on a few limitations throughout the current systematic review. Different studies employed different diagnostic criteria for their samples, causing increased heterogeneity and decreased comparability among studies. Furthermore, conducting more studies with larger sample sizes seems necessary to yield more statistically significant outcomes. Notably, when a single gene affects multiple phenotypes and causes various diseases, the phenomenon is called pleiotropy.^106^ Concerning similar immune pathways of periodontitis and PI, it seems logical to assert that gene polymorphisms associated with periodontitis may be linked to PI development. Thus, evaluating periodontitis-associated genetic variations among PI patients would help define which polymorphisms should be investigated in future research.

 Increasing the knowledge of genetic variations and their association with PIM and PI among clinicians would help them select the most suitable treatment plan, predict the prognosis more accurately, and avoid ineffective interventions when facing peri-implant complications. Nevertheless, further research among different populations is required to introduce particular gene polymorphisms as absolute risk factors for PIM and PI.

## Conclusion

 According to the present meta-analysis, polymorphisms in the genotype of IL-1α -889, IL-1β + 3954, and OPG -3618 might be associated with PI development.

## Competing Interests

 The authors declare that they have no competing interests.

## Consent for Publication

 Not applicable.

## Data Availability Statement

 The data from the reported study are available upon request from the corresponding author.

## Ethical Approval

 Not applicable.

## Supplementary Files


Supplementary file 1 contains Tables S1-S4 and Figures S1-S7.
