# Supplementary material for: The association of gene polymorphisms with peri-implant mucositis and peri-implantitis: A systematic review and meta-analysis
Source: J Adv Periodontol Implant Dent. 2025 Jan 6;17(2):77–89. doi: 10.34172/japid.2025.3432 (PMC12261067; doi:10.34172/japid.2025.3432)
Supplement: Supplementary file 1 — contains Tables S1-S4 and Figures S1-S7. [file japid-17-77-s001.pdf]

Shahbazi et al, *J Adv Periodontol Implant Dent.* 2025;17(2):77-S1
doi: 10.34172/japid.2025.3432
https://japid.tbzmed.ac.ir

Supplementary file 1

Table S1. Demographic characteristics of included studies

Author & Year	Study Design	Country	Number of Patients			Age			Gender			Number of Smokers		
			PI	PIM	H	PI	PIM	H	PI	PIM	H	PI	PIM	H
Cardoso et al. (2022) [25]	Case-Control	Portugal	10	0	10	62±11	-	54±22	6 M 4 F	-	3 M 7 F	3	-	3
Chang et al. (2021) [33]	Case-Control	China	150	0	150	43.37±6.25	-	42.55±6.93	79 M 71 F	-	83 M 67 F	68	-	69
Chen & Chen (2021) [34]	Case-Control	China	162	0	162	NM			89 M 73 F	-	84 M 78 F	73	-	75
Qi et al. (2021) [35]	Case-Control	China	127	0	133	44.07±6.05	-	43.42±6.31	70 M 57 F	-	72 M 61 F	60	-	64
Saremi et al. (2021) [13]	Case-Control	Iran	50	0	89	42.2±12.2	-	40.4±13.5	24 M 26 F	-	43 M 46 F	NM		
He et al. (2020) [36]	Case-Control	China	144	0	174	NM			88 M 56 F	-	92 M 82 F	0	-	0
Silva et al. (2020) [26]	Case-Control	Brazil	13	30	71	50±14.1	51±12.5	35.7±13	6 M 7 F	12 M 18 F	28 M 43 F	7	2	10
Saremi et al. (2019) [37]	Case-Control	Iran	50	0	90	56.2	-	42.4	22 M	-	44 M	0	-	0

									28 F		46 F			
Petkovic-Curcin et al. (2017) [38]	Case-Control	Serbia	34	0	64	58	-	58	26 M<br>8 F	-	44 M<br>20 F	24	-	27
Goncalves et al. (2016) [17]	Case-Control	Brazil	28	0	72	54.5±12.30	-	51.7±14.4	7 M<br>21 F	-	27 M<br>45 F	1	-	7
Kadkhodazadeh et al. (2016) [18]	Case-Control	Iran	38	0	84	50.2	-	38.4	18 M<br>20 F	-	43 M<br>41 F	0	-	0
Zhou & Zhao (2016) [39]	Case-Control	China	110	0	116	42.85±11.21	-	43.02±10.94	89 M<br>21 F	-	94 M<br>22 F	NM		
Coelho et al. (2016) [40]	Case-Control	Brazil	86	0	129	57.89±11.29	-	53.27±13.18	24 M<br>62 F	-	43 M<br>86 F	8	-	13
Casado et al. (2015) [41]	Cross-Sectional	Brazil	34	0	93	51.2±13.3	-	55.1±11.8	10 M<br>24 F	-	32 M<br>61 F	1	-	7
Garcia-Delaney et al. (2015) [42]	Case-Control	Spain	27	0	27	54.4	-	50.6	9 M<br>18 F	-	NM	<10 cig/day: 7<br>≥10 cig/day: 20	-	<10 cig/day: 5<br>≥10 cig/day: 22
Kadkhodazadeh et al. (2014) [15]	Case-Control	Iran	38	0	81	50.2	-	38.4	20 M<br>18 F	-	41 M<br>40 F	0	-	0
Ebadian et al. (2014) [19]	Cross-sectional	Iran	43	0	86	40	-	44	21 M<br>22 F	-	41 M<br>42 F	0	-	0
Casado et al. (2013) [43]	Case-Control	Brazil	31	20	52	53±3.7	NM	47.4±8.7	12 M<br>19 F	NM	22 M<br>30 F	0	0	0

Kadkhodazadeh et al. (2013) [20]	Cross-sectional	Iran	37	0	83	50.2	-	38.4	19 M 18 F	-	40 M 43 F	0	-	0
Kadkhodazadeh et al. (2013) [44]	Cross-sectional	Iran	38	0	84	NM			NM			0	-	0
Kadkhodazadeh et al. (2013) [21]	Cross-sectional	Iran	38	0	82	50.2	-	45.4	20 M 18 F	-	39 M 43 F	0	-	0
Kadkhodazadeh et al. (2013) [45]	Cross-sectional	Iran	38	0	84	32 to 58	-	31 to 84	26 M 12 F	-	44 M 40 F	0	-	0
Kadkhodazadeh et al. (2013) [14]	Cross-sectional	Iran	37	0	81	50.2	-	38.4	18 M 19 F	-	41 M 40 F	Smokers were included but their exact number was not mentioned		
Kadkhodazadeh et al. (2012) [16]	Cross-sectional	Iran	40	0	89	58.3	-	40.4	19 M 21 F	-	43 M 46 F	0	-	0
Kadkhodazadeh et al. (2012) [22]	Cross-sectional	Iran	30	0	48	50.2	-	38.4	16 M 14 F	-	27 M 21 F	0	-	0
Melo et al. (2012) [46]	Case-Control	Brazil	16	0	31	51.1±3.1	-	45.2±3.4	15 M 34 F	-	17 M 24 F	0	-	0
Hamdy & Ebrahem (2011) [23]	Case-Control	Egypt	25	0	25	43±5.51	-	38.5±5.4	20 M 5 F	-	18 M 7 F	0	-	0
Cury et al. (2009) [47]	Case-Control	Brazil	41	0	49	48.9±3.1	-	42.8±2.9	7 M 10 F	-	9 M 10 F	0	-	0
Cury et al. (2007) [48]	Case-Control	Brazil	17	0	19	68	-	66	24 M 47 F	-	23 M 26 F	76%	-	49%
Laine et al. (2006) [49]	Case-Control	Sweden	71	0	49	NM			NM			NM		

PI = Peri-implantitis, PIM = Peri-implant mucositis, H = Healthy, M = Male, F = Female, NM = Not mentioned

Table S2. Study characteristics and summary of findings

Author & Year	Investigated Polymorphism	Diagnostic Criteria	Sample Site	Outcome
Cardoso et al. (2022) [25]	IL-1α -889<br>IL-1β +3954	PI:<br>BoP and/or suppuration<br>PD ≥6 mm<br>MBL ≥3 mm	Jugal mucosa	There was no statistically significant difference in the proportions of IL-1 gene polymorphisms between the health and disease groups.
Chang et al. (2021) [33]	EGF (rs2237051)<br>EGF (rs4444903)	PI:<br>BoP with/without suppuration<br>PD>5 mm<br>At least one site with MBL exposing two edges	Venous blood	EGF (rs2237051) gene polymorphisms were related to PI susceptibility. The GG genotype and G allele might be protective factors for the onset of PI.
Chen & Chen (2021) [34]	IL-16 (rs11556218)<br>IL-16 (rs4072111)	PI:<br>PD ≥6 mm<br>Excessive BoP<br>Distance between bone crest and implant shoulder ≥3 mm	Buccal mucosa	The CT genotype of the IL-16 gene (rs4072111) SNP can be used as a factor for assessing PI risk.
Qi et al. (2021) [35]	CXCR2 (rs2230054)<br>CXCR2 (rs1126580)	PI:<br>BoP<br>PD>4 mm<br>At least one area with MBL exposing 2 edges	Buccal epithelial cell	The CT genotype of (rs2230054) and the AG genotype and G allele of (rs1126580) serve as risk factors for the occurrence of PI.
Saremi et al. (2021) [13]	IL-10 -819<br>IL-10 -592<br>IL-1β +3954<br>TNFα-308<br>TNFα -857	PI:<br>PD>5 mm<br>BoP with/without Pus<br>At least one site with≥2 mm MBL	Venous blood	Specific gene polymorphisms of IL-10 -819 C/T, IL-10 -592 C/A, and IL-1ß + 3954 C/T may play a role in the pathogenesis of PI and increase its risk of occurrence.
He et al. (2020) [36]	TNF-α -308 (rs1800629)<br>IL-1α -889 (rs1800587)<br>IL-1β +3954 (rs1143634)	PI:<br>PD≥4 mm<br>BoP<br>Positive GI<br>Positive plaque index<br>≥2 threads MBL	Buccal epithelial cell	The IL-1α − 889C/T or IL-1β + 3954C/T genetic polymorphisms were associated with the risk of PI.
Silva et al. (2020) [26]	RANK (rs3826620)	NM	Saliva	The studied genetic polymorphism in RANK, RANKL, and OPG was not associated with PIM and PI in a Brazilian population from the Amazon region.

	RANKL (rs9594738)<br>OPG (rs2073618)			
Saremi et al. (2019) [37]	FCγR IIIa<br>FCγR IIa<br>FCγR IIIb	PI:<br>PD>5<br>BoP with/without pus<br>At least one site with≥2 mm MBL and exposing ≥2 threads<br>ISI V, VI, and VII	Venous blood	The FCGRIIa (rs1801274), FCGRIIIa (rs396991), and FCGRIIIb (rs1050501) polymorphisms were significantly associated with PI and may have a role in the pathogenesis of the disease.
Petkovic-Curcin et al. (2017) [38]	IL-10 -1082<br>TNFα -308<br>IL-6 -174<br>CD14 -159<br>IL-1ra	PI:<br>PD≥4 mm<br>BoP<br>Positive GI<br>Positive plaque index<br>MBL exposing≥2 threads	Peripheral blood	The findings suggest that smoking and the presence of TNFα -308 GA/AA genotypes may increase the risk for PI, while CD14-159 polymorphic CT/TT genotypes decrease the risk.
Goncalves et al. (2016) [17]	MMP-13 (rs2252070)<br>TGFB3 (rs2268626)<br>TIMP2 (rs7501477)	PI:<br>MBL>1 mm during the first year and>0.2 mm per year	Saliva	There is no association between PI and polymorphisms in the MMP13, TIMP2, and TGFB3 genes.
Kadkhodazadeh et al. (2016) [18]	NRAMP1 (rs17235409)<br>NRAMP1 (rs2276631)	PI:<br>PD≥5 mm with/without suppuration/BoP<br>Plaque index>20%<br>MBL expose ≥2 threads<br>ISI VI, VII, and VIII	Venous blood	Distribution of genotypes differed insignificantly in comparison of PI and control groups for rs2276631 and either rs17235409 polymorphisms.
Zhou & Zhao (2016) [39]	OPG (rs2073617)<br>OPG (rs2073618)	PI:<br>No loosening<br>Swelling of mucosa<br>BoP<br>MBL>3mm	Venous blood	OPG rs2073618 polymorphism may be related to the risk of PI, but not rs2073617
Coelho et al. (2016) [40]	BMP4<br>FGF3<br>FGF10<br>FGFR1	PI:<br>MBL>1 mm and>0.2 mm per year	Buccal epithelial cell	The TT polymorphic genotype for BMP4 (rs2761884) was associated with healthy peri-implant. FGF3 (rs4631909) (TT+CT genotype) also showed an association with the control group. The frequency of the C allele for FGF3 (rs4631909) showed a tendency for association with PI. FGF10 CCTG, BMP4 GAAA, and GGGA haplotypes were associated with PI.

Casado et al. (2015) [41]	BRINP3 (rs1342913)<br>BRINP (rs1935881)	PI:<br>MBL>1 mm and>0.2 per year	Buccal epithelial cell	The BRINP3 polymorphic variant (rs1342913) is associated with PI.
Garcia-Delaney et al. (2015) [42]	IL-1α -889<br>IL-1β +3953<br>IL-1RN+2018	PI:<br>BoP or suppuration<br>MB>2mm	Oral mucosa	IL-1 genotypes do not seem to be good predictors of PI in the great majority of smoking patients.
Kadkhodazadeh et al. (2014) [15]	RANK (rs35211496)<br>RANK (rs3018362)	PI:<br>PD>5 with/without suppuration<br>MBL<br>ISI VI, VII, and VIII	Venous blood	The CC genotype of the rs35211496 RANK gene polymorphism was significantly associated with PI.
Ebadian et al. (2014) [19]	Hp 2-2	PI:<br>PD>5 mm<br>BoP with/without suppuration<br>MBL<br>ISI VI, VII, and VIII	Venous blood	Hp polymorphisms may not play a role in the development of PI among Iranians.
Casado et al. (2013) [43]	IL-6 -174	PI:<br>Mobility<br>Suppuration<br>MBL<br><br>PIM:<br>BoP<br>red mucosa<br>swelling	Mouth wash	The frequency of the genotype IL-6174 GG and the allele G was different among healthy and diseased groups.
Kadkhodazadeh et al. (2013) [20]	IL-17R (rs879576)	PI:<br>PD>5 mm<br>BoP with/without suppuration<br>MBL exposing at least 2 threads<br>ISI VI, VII, and VIII	Venous blood	This article demonstrates that polymorphism of IL-17R plays no significant role in the incidence of PI.
Kadkhodazadeh et al. (2013) [44]	IL-17 (rs10484879)	PI:<br>PD>5 mm<br>BoP with/without suppuration<br>MBL exposing at least 2 threads<br>ISI VI, VII, and VIII	Venous blood	The CC genotype of IL17 polymorphism (rs10484879) may contribute to the pathogenesis of PI.

Kadkhodazadeh et al. (2013) [21]	BRAF (rs10487888)	PI: PD>5 mm BoP with/without suppuration MBL exposing at least 2 threads ISI VI, VII, and VIII	Venous blood	The BRAF gene polymorphism (rs10487888) may not be a genetic determinant for increasing the risk of PI among the Iranian population.
Kadkhodazadeh et al. (2013) [45]	MiR146a (rs2910146) MiR499 (rs3746444)	PI: PD>5 mm BoP with/without suppuration MBL exposing at least 2 threads ISI VI, VII, and VIII	Venous blood	MiR146a (rs2910146) and MiR499 (rs3746444) gene polymorphisms may be genetic determinants for the increased risk of PI in Iranians.
Kadkhodazadeh et al. (2013) [14]	RANKL (rs9533156) RANKL (rs2277438)	PI: PD>5 mm BoP with/without suppuration MBL exposing at least 2 threads ISI VI, VII, and VIII	Venous blood	The results of this study indicate that the CT genotype of rs9533156 RANKL gene polymorphism was significantly associated with PI.
Kadkhodazadeh et al. (2012) [16]	OPG -950 (rs2073617) OPG -1181 (rs2073618)	PI: PD>5 mm BoP with/without suppuration MBL exposing at least 2 threads ISI VI, VII, and VIII	Venous blood	Our results indicate that a SNP at G1181C is associated with the presence of PI.
Kadkhodazadeh et al. (2012) [22]	TANK (rs3820998) TANK (rs1921310)	PI: PD>5 mm BoP with/without suppuration MBL exposing at least 2 threads ISI VI, VII, and VIII	Venous blood	It seems that these two polymorphisms do not play a significant role in the pathogenesis of PI among the Iranian population.
Melo et al. (2011) [46]	IL-1β -511 IL-1β +3954 IL-6 -174	PI: PD≥ 5 mm BoP with/without suppuration MBL>3mm	Oral mucosa	The studied gene polymorphisms did not influence PID.

Hamdy & Ebrahem (2011) [23]	IL-1α -889<br>IL-1β +3954	PI:<br>PD>4 mm<br>MBL<br>modified GI>1.5<br>modified Plaque index>1.5	Oral mucosa	The combination of IL-1 allele 2 (IL-1α -889 and IL-1β+3954) in patients with inflamed periodontal or peri-implant tissues may act as a risk factor that increases tissue destruction. IL-1 gene polymorphism may have a negative effect on treatment outcomes of PI in genotype-positive individuals.
Cury et al. (2009) [47]	TNF-α -308	PI:<br>MBL> one-third of implant height<br>suppuration/BoP	Oral mucosa	Polymorphism of the TNFa-308 gene was not associated with an increased risk of PI in the population evaluated in this study.
Cury et al. (2007) [48]	TNF-α -308	PI:<br>MBL>3 threads on Branemark implant<br>BoP and/or suppuration	Mouth rinse	Polymorphism in allele 2 of the TNF-α -308 gene is not associated with an increased risk for peri-implant bone loss following prosthetic reconstruction.
Laine et al. (2006) [49]	IL-1α -889<br>IL-1β +3954<br>IL-1β -511<br>IL-1RN (VNTR)	NM	NM	IL-1RN gene polymorphism is associated with PI and may represent a risk factor for this disease.

PI = Peri-implantitis, PD = Pocket Depth, BoP = Bleeding on probing, MBL = Marginal bone loss, IL = Interleukin, MBL = Marginal bone loss, EGF = Epidermal growth factor, SNP = Single nucleotide polymorphism, CXCR = CXC chemokine receptor, TNF = Tumor necrosis factor, GI = Gingival index, RANK = Receptor activator of nuclear factor κ β, RANKL = receptor activator of nuclear factor κ β ligand, OPG = Osteoprotegerin, NM = Not mentioned, PIM = Peri-implant mucositis, ISI = Implant success index, CD = Cluster of differentiation, MMP = Matrix metalloproteinase, TGF = Transforming growth factor, TIMP = Tissue inhibitors of metalloproteinases, NRAMP = Natural resistance-associated macrophage proteins, BMP = Bone morphogenic protein, FGF = Fibroblast growth factor, BRINP = Bone morphogenic protein/retinoic acid inducible neural specific, Hp = Haptolglobin, MiR = MicroRNA, TANK = TNFR-associated factor family member-associated NF-κ β, PID = Peri-implant disease, VNTR = Variable number tandem repeat

Table S3: Study characteristics and summary of findings.

Supplementary File 3: Study Characteristics and Summary of Findings

**Author & Year**	**History of Periodontal Disease**			**Plaque Index**			**Position of Diseased Implant**			**Platform Type**			**Thin Soft Tissue Biotype**			**Restoration Type**	**Loading Time**
	**PI**	**PIM**	**H**	**PI**	**PIM**	**H**	**PI**	**PIM**	**H**	**PI**	**PIM**	**H**	**PI**	**PIM**	**H**		
Cardoso et al. (2022) [25]	8	-	5	NM			NM			NM			NM			NM	12 m
Chang et al. (2021) [33]	84	-	68	2.27±0.61	-	2.27±0.61	81 anterior<br>69 posterior	-	96 anterior<br>54 posterior	63 external hex<br>34 internal hex<br>45 morse cone<br>8 others	-	70 external hex<br>22 internal hex<br>48 morse taper<br>10 others	46.67 %	-	56.67 %	NM	12 m
Chen & Chen (2021) [34]	90	-	73	2.23±0.66	-	0.87±0.62	87 anterior<br>75 posterior	-	104 anterior<br>58 posterior	68 external hex<br>37 internal hex<br>49 morse cone<br>8 others	-	76 external hex<br>24 internal hex<br>52 morse taper<br>10 others	46.91 %	-	54.94 %	NM	min 12 m

Qi et al. (2021) [35]	73	-	59	2.35±0.35	-	0.83±0.44	71 anterior<br><br>56 posterior	-	89 anterior<br><br>44 posterior	56 external hex<br><br>28 internal hex<br><br>37 morse cone<br><br>6 others	-	64 external hex<br><br>20 internal hex<br><br>40 morse cone<br><br>9 others	48%	-	54.9 %	NM	min 12 m
Saremi et al. (2021) [13]	0	-	0	NM			NM			NM			NM			NM	min 12 m
He et al. (2020) [36]	78	-	58	2.37±0.6	-	0.83±0.78	67 anterior<br><br>77 posterior	-	91 anterior<br><br>83 posterior	70 external hex<br><br>22 internal hex<br><br>42 morse cone<br><br>10 others	-	76 external hex<br><br>39 internal hex<br><br>51 morse cone<br><br>8 others	54.9 %	-	44.8 %	NM	NM
Silva et al. (2020) [26]	9	13	18	NM			NM			NM			61.55 %	53.3 %	21.1 %	NM	NM
Saremi et al. (2019) [37]	0	-	0	NM			NM			NM			NM			NM	min 12 m
Petkovic-Curcin et al. (2017) [38]	21	-	13	NM			NM			Platform switched			NM			Fixed cemented prostheses	min 12 m

Goncalves et al. (2016) [17]	0	-	0	NM			17 maxilla<br><br>11 mandible	-	41 maxilla<br><br>31 mandible	16 External hex<br><br>1 Internal hex<br><br>10 Morse cone<br><br>1 Others	-	35 External hex<br><br>5 Internal hex<br><br>29 Morse cone<br><br>3 Others	39.2 %	-	40.2 %	NM	PI: 31.17±25.21<br><br>H: 34.71±31.62
Kadkhodazadeh et al. (2016) [18]	No current periodontal disease			NM			NM			NM			NM			NM	12 m
Zhou & Zhao (2016) [39]	NM			NM			NM			NM			NM			NM	NM
Coelho et al. (2016) [40]	No untreated periodontitis			NM			43 maxilla<br><br>43 mandible		63 maxilla<br><br>68 mandible	50 external hex<br><br>8 internal hex<br><br>27 morse cone<br><br>1 others	-	66 external hex<br><br>10 internal hex<br><br>55 morse cone<br><br>5 others	51.16 %	-	37.20 %	NM	PI: 35.85<br>H: 33.95
Casado et al. (2015) [41]	0	-	0	0.17±0.38	-	0.03±0.18	64 maxilla<br><br>56 mandible	-	122 maxilla<br><br>102 mandible	21 external hex	-	44 external hex	38.2 %	-	37.6 %	Single crown<br><br>Short-span fixed partial denture	PI: 31.7±23.7<br><br>H:

										2 internal hex<br><br>10 morse cone<br><br>1 others		8 internal hex<br><br>35 morse cone<br><br>6 others					35.33±34.01 (min 6 m)
Garcia-Delaney et al. (2015) [42]	26	-	19	NM			1 anterior<br><br>11 posterior<br><br>15 both	-	4 anterior<br><br>10 posterior<br><br>13 both	NM			NM			Partial, total, or removable	18 m
Kadkhodazadeh et al. (2014) [15]	0	-	0	NM			NM			NM			NM			NM	12 m
Ebadian et al. (2014) [19]	0	-	0	NM			NM			NM			NM			NM	12 m
Casado et al. (2013) [43]	20	11	12	NM			94 maxilla<br><br>77 mandible	-	57 maxilla<br><br>67 mandible	NM			NM			Single crown<br><br>Short-span fixed partial denture	12 to 60 m
Kadkhodazadeh et al. (2013) [20]	0	-	0	NM			NM			NM			NM			NM	12 m
Kadkhodazadeh et al. (2013) [44]	0	-	0	NM			NM			NM			NM			NM	12 m
Kadkhodazadeh et al. (2013) [21]	0	-	0	NM			NM			NM			NM			NM	12 m
Kadkhodazadeh et al. (2013) [45]	0	-	0	NM			NM			NM			NM			NM	12 m

Kadkhodazadeh et al. (2013) [14]	0	-	0	NM			NM			NM	NM	NM	NM
Kadkhodazadeh et al. (2012) [16]	0	-	0	NM			NM			NM	NM	NM	36 m
Kadkhodazadeh et al. (2012) [22]	NM			NM			NM			NM	NM	NM	12 m
Melo et al. (2011) [46]	NM			NM			24 maxilla<br><br>25 mandible	-	24 maxilla<br><br>27 mandible	NM	NM	NM	6 to 144 m
Hamdy & Ebrahem (2011) [23]	NM			2.51±0.41	-	1.36±0.06	NM			NM	NM	NM	NM
Cury et al. (2009) [47]	NM			NM			10 maxilla<br><br>9 mandible	-	10 maxilla<br><br>7 mandible	NM	NM	NM	6 to 31 m
Cury et al. (2007) [48]	56.30 % toot loss due to periodontitis	-	75.50 % tooth loss due to periodontitis	NM			NM			NM	NM	Fixed prostheses	24 m
Laine et al. (2006) [49]	NM			NM			NM			NM	NM	NM	NM

PI = Peri-implantitis, PIM = Peri-implant mucositis, H = Healthy, NM = Not mentioned, min = Minimum, m = Month

Table S4: Quality Assessment Using "Suggested Guidelines for Systematic Reviews of Periodontal Genetic Association Studies"

Authors & Year	Selection (4 items)	Comparability (1 item)	Exposure (3 items)	Study Methodology/Design (4 items)	Genetic Analysis (8 items)	Total (20 items)
Cardoso et al. (2022) [25]	✓✓✓	✓	✓✓✓	✓✓	✓✓✓✓	13
Chang et al. (2021) [33]	✓✓✓		✓✓✓	✓	✓✓✓✓✓	12
Chen & Chen (2021) [34]	✓✓✓		✓✓	✓✓	✓✓✓✓✓	12
Qi et al. (2021) [35]	✓✓✓		✓✓✓	✓✓	✓✓✓✓✓	13
Saremi et al. (2021) [13]	✓✓✓		✓✓✓	✓✓	✓✓✓✓✓	13
He et al. (2020) [36]	✓✓✓		✓✓	✓✓✓	✓✓✓	11
Silva et al. (2020) [26]	✓✓		✓✓✓	✓✓	✓✓✓✓	11
Saremi et al. (2019) [37]	✓✓✓		✓✓✓	✓	✓✓✓✓✓	12
Petkovic-Curcin et al. (2017) [38]	✓✓✓	✓	✓✓	✓✓	✓✓✓	11
Goncalves et al. (2016) [17]	✓		✓✓✓	✓✓	✓✓✓✓✓	11
Kadkhodazadeh et al. (2016) [18]	✓✓✓		✓✓✓	✓	✓✓✓✓	11
Zhou & Zhao (2016) [39]	✓		✓✓	✓✓	✓✓✓✓✓	10
Coelho et al. (2016) [40]	✓		✓✓✓	✓✓	✓✓✓✓✓	11
Casado et al. (2015) [41]	✓	✓	✓✓✓	✓✓	✓✓✓✓	11
Garcia-Delaney et al. (2015) [42]	✓✓✓✓		✓✓✓	✓✓✓✓	✓	12
Kadkhodazadeh et al. (2014) [15]	✓✓✓		✓	✓✓	✓✓✓✓✓	11
Ebadian et al. (2014) [19]	✓✓✓		✓✓✓		✓✓✓✓✓	11
Casado et al. (2013) [43]	✓		✓✓✓	✓✓	✓✓✓✓	10
Kadkhodazadeh et al. (2013) [20]	✓✓✓		✓✓✓		✓✓✓✓✓	11
Kadkhodazadeh et al. (2013) [44]	✓✓✓		✓✓✓	✓	✓✓✓✓✓	12
Kadkhodazadeh et al. (2013) [21]	✓✓✓		✓✓✓	✓	✓✓✓✓	11
Kadkhodazadeh et al. (2013) [45]	✓✓✓		✓✓✓	✓	✓✓✓✓	11
Kadkhodazadeh et al. (2013) [14]	✓✓✓		✓✓✓	✓	✓✓✓✓✓	12
Kadkhodazadeh et al. (2012) [16]	✓✓✓		✓✓✓	✓✓	✓✓✓✓	12
Kadkhodazadeh et al. (2012) [22]	✓✓✓		✓✓✓	✓	✓✓✓✓	11
Melo et al. (2011) [46]	✓✓		✓✓	✓	✓✓✓✓✓	10
Hamdy & Ebrahem (2011) [23]	✓✓		✓✓✓	✓	✓✓	8
Cury et al. (2009) [47]	✓✓✓		✓✓✓	✓	✓✓✓✓	11

Cury et al. (2007) [48]	✓✓		✓✓	✓	✓	6
Laine et al. (2006) [49]	✓✓	✓	✓✓✓	✓✓	✓✓✓✓	12

Figure S1. The meta-analysis for the association between IL-1α -889 allelic frequency and PI risk. (a) C allele (b) T allele.

(a)

Study name	Odds ratio	Lower limit	Upper limit	Z-Value	p-Value
Cardoso et al. 2022	0.412	0.087	1.952	-1.118	0.264
He et al. 2020	0.441	0.301	0.647	-4.191	0.000
	0.439	0.303	0.637	-4.337	0.000

Statistics for each study

Odds ratio and 95% CI

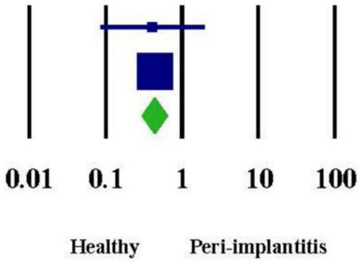

Fixed-effect model, Heterogeneity: Tau-squared=0, I-squared=0%, P=0.933

(b)

Study name	Odds ratio	Lower limit	Upper limit	Z-Value	p-Value
Cardoso et al. 2022	2.429	0.512	11.511	1.118	0.264
He et al. 2020	2.268	1.546	3.326	4.191	0.000
Laine et al. 2006	1.023	0.477	2.196	0.058	0.953
	1.953	1.398	2.729	3.925	0.000

Statistics for each study

Odds ratio and 95% CI

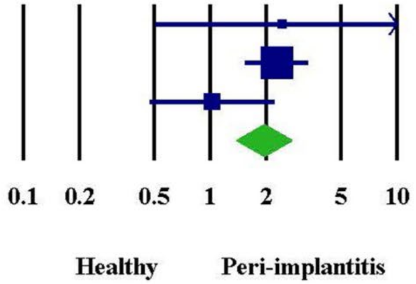

Fixed-effect model, Heterogeneity: Tau-squared=0.10, I-squared=41.44%, P=0.181

Figure S2. The meta-analysis for the association between IL-1β +3954 allelic frequency and PI risk. (a) T allele.

Study name	Odds ratio	Lower limit	Upper limit	Z-Value	p-Value
	Statistics for each study				
Cardoso et al. 2022	4.846	0.863	27.221	1.792	0.073
Saremi et al. 2021	6.107	2.481	15.032	3.937	0.000
He et al. 2020	1.805	1.257	2.592	3.201	0.001
Melo et al. 2011	1.967	0.795	4.869	1.463	0.143
Laine et al. 2006	0.915	0.413	2.028	-0.219	0.827
	2.195	1.207	3.993	2.575	0.010

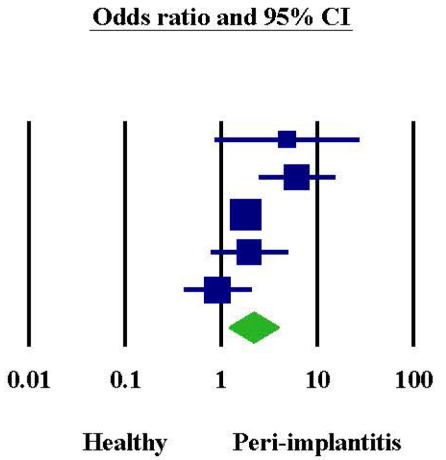

Random-effects model, Heterogeneity: Tau-squared=0.26, I-squared=63.28%, P=0.028

Figure S3. The meta-analysis for the association between OPG -3618 allelic frequency and PI risk. (a) C allele.

Study name	Odds ratio	Lower limit	Upper limit	Z-Value	p-Value
Silva et al. 2020	1.519	0.878	2.626	1.495	0.135
Zhou & Zhao 2016	1.467	1.011	2.131	2.016	0.044
	1.483	1.090	2.019	2.507	0.012

(Statistics for each study)

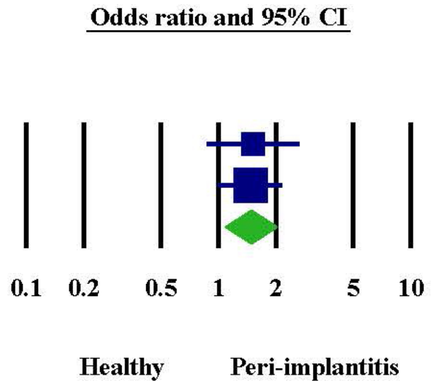

Fixed-effect model, Heterogeneity: Tau-squared=0, I-squared=0%, P=0.919

Figure S4. The meta-analysis for the association between IL-1β -511 gene polymorphism and PI risk. (a) CC genotype. (b) CT genotype. (c) TT genotype. (d) C allele.

(a)

Study name	Odds ratio	Lower limit	Upper limit	Z-Value	p-Value
	**Statistics for each study**				
Melo et al. 2011	0.700	0.180	2.725	-0.514	0.607
Laine et al. 2006	1.155	0.550	2.429	0.381	0.703
	1.030	0.536	1.976	0.088	0.930

Odds ratio and 95% CI

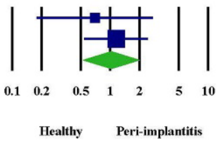

Fixed-effect model, Heterogeneity: Tau-squared=0, I-squared=0%, P=0.526

(b)

Study name	Odds ratio	Lower limit	Upper limit	Z-Value	p-Value
	**Statistics for each study**				
Melo et al. 2011	1.204	0.349	4.152	0.293	0.769
Laine et al. 2006	0.744	0.358	1.545	-0.793	0.428
	0.842	0.449	1.580	-0.534	0.593

Odds ratio and 95% CI

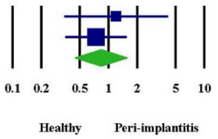

Fixed-effect model, Heterogeneity: Tau-squared=0, I-squared=0%, P=0.512

(c)

Study name	Odds ratio	Lower limit	Upper limit	Z-Value	p-Value
	**Statistics for each study**				
Melo et al. 2011	1.277	0.401	4.073	0.414	0.679
Laine et al. 2006	1.333	0.199	8.921	0.297	0.767
	1.292	0.480	3.477	0.508	0.612

Odds ratio and 95% CI

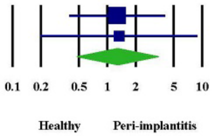

Fixed-effect model, Heterogeneity: Tau-squared=0, I-squared=0%, P=0.970

(d)

Study name	Odds ratio	Lower limit	Upper limit	Z-Value	p-Value
	**Statistics for each study**				
Melo et al. 2011	0.812	0.342	1.929	-0.472	0.637
Laine et al. 2006	2.425	1.150	5.114	2.327	0.020
	1.436	0.492	4.190	0.662	0.508

Odds ratio and 95% CI

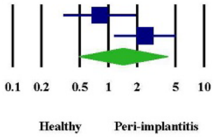

Random-effects model, Heterogeneity: Tau-squared=0.42, I-squared=71.59%, P=0.061

Figure S5. The meta-analysis for the association between IL-6 -174 gene polymorphism and PI risk. (a) GG genotype. (b) CG genotype. (c) CC genotype. (d) G allele.

(a)

Study name	Odds ratio	Lower limit	Upper limit	Z-Value	p-Value
Pektovic-Curcin 2017	34.103	1.994	583.259	2.436	0.015
Casado et al. 2013	1.847	0.747	4.568	1.329	0.184
Melo et al. 2011	0.612	0.177	2.120	-0.774	0.439
	2.091	0.446	9.811	0.935	0.350

Statistics for each study — Odds ratio and 95% CI

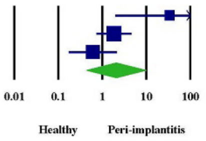

Random-effects model, Heterogeneity: Tau-squared=1.22, I-squared=70.78%, P=0.033

(b)

Study name	Odds ratio	Lower limit	Upper limit	Z-Value	p-Value
Casado et al. 2013	0.693	0.277	1.735	-0.782	0.434
Melo et al. 2011	1.901	0.542	6.675	1.003	0.316
	0.985	0.470	2.065	-0.041	0.968

Statistics for each study — Odds ratio and 95% CI

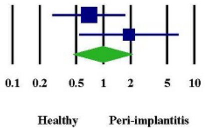

Fixed-effect model, Heterogeneity: Tau-squared=0.19, I-squared=38.12%, P=0.204

(c)

Study name	Odds ratio	Lower limit	Upper limit	Z-Value	p-Value
Casado et al. 2013	0.313	0.035	2.815	-1.036	0.300
Melo et al. 2011	0.616	0.024	15.989	-0.291	0.771
	0.387	0.063	2.390	-1.022	0.307

Statistics for each study — Odds ratio and 95% CI

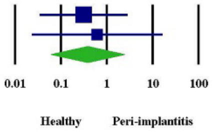

Fixed-effect model, Heterogeneity: Tau-squared=0, I-squared=0%, P=0.736

(d)

Study name	Odds ratio	Lower limit	Upper limit	Z-Value	p-Value
Casado et al. 2013	1.752	0.838	3.664	1.489	0.136
Melo et al. 2011	0.770	0.266	2.227	-0.482	0.630
	1.341	0.731	2.457	0.948	0.343

Statistics for each study — Odds ratio and 95% CI

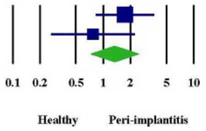

Fixed-effect model, Heterogeneity: Tau-squared=0.12, I-squared=35.55%, P=0.213

Figure S6. The meta-analysis for the association between OPG -3617 gene polymorphism and PI risk. (a) CC genotype. (b) CT genotype. (c) TT genotype.

(a)

Study name	Odds ratio	Lower limit	Upper limit	Z-Value	p-Value
Zhou & Zhao 2016	1.810	0.833	3.930	1.499	0.134
Kadkhodazadeh et al. 2012	1.569	0.518	4.751	0.796	0.426
	1.727	0.915	3.259	1.685	0.092

Statistics for each study

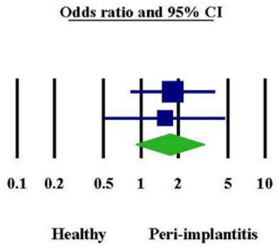

Fixed-effect model, Heterogeneity: Tau-squared=0, I-squared=0%, P=0.836

(b)

Study name	Odds ratio	Lower limit	Upper limit	Z-Value	p-Value
Zhou & Zhao 2016	0.864	0.513	1.457	-0.547	0.585
Kadkhodazadeh et al. 2012	0.544	0.255	1.161	-1.573	0.116
	0.745	0.484	1.145	-1.343	0.179

Statistics for each study

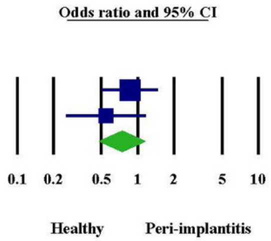

Fixed-effect model, Heterogeneity: Tau-squared=0, I-squared=0%, P=0.325

(c)

Study name	Odds ratio	Lower limit	Upper limit	Z-Value	p-Value
Zhou & Zhao 2016	0.870	0.508	1.489	-0.509	0.611
Kadkhodazadeh et al. 2012	1.052	0.510	2.170	0.138	0.890
	0.931	0.604	1.433	-0.326	0.744

Statistics for each study

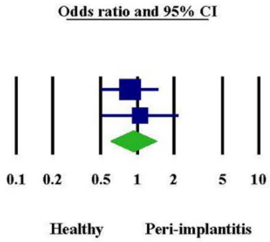

Fixed-effect model, Heterogeneity: Tau-squared=0, I-squared=0%, P=0.678

Figure S7. The meta-analysis for the association between TNF-α -308 gene polymorphism and PI risk. (a) GG genotype. (b) AG genotype. (c) AA genotype. (d) A allele.

(a)

Study name	Odds ratio	Lower limit	Upper limit	Z-Value	p-Value
Saremi et al. 2021	1.848	0.442	7.734	0.841	0.401
He et al. 2020	0.699	0.397	1.232	-1.238	0.216
Petkovic-Curcin et al. 2017	0.113	0.041	0.308	-4.262	0.000
Cury et al. 2009	0.731	0.287	1.865	-0.655	0.512
Cury et al. 2007	0.381	0.088	1.650	-1.290	0.197
	0.513	0.224	1.174	-1.580	0.114

Statistics for each study

Odds ratio and 95% CI

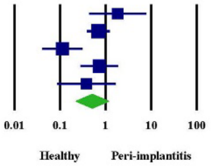

Random-effects model, Heterogeneity: Tau-squared=0.59, I-squared=70.60%, P=0.009

(b)

Study name	Odds ratio	Lower limit	Upper limit	Z-Value	p-Value
Saremi et al. 2021	1.246	0.543	2.856	0.519	0.604
He et al. 2020	1.117	0.477	2.611	0.254	0.799
Cury et al. 2009	1.194	0.429	3.322	0.340	0.734
Cury et al. 2007	2.909	0.597	14.185	1.321	0.186
	1.290	0.791	2.102	1.021	0.307

Statistics for each study

Odds ratio and 95% CI

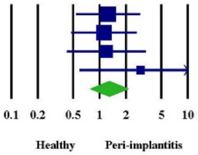

Fixed-effect model, Heterogeneity: Tau-squared=0, I-squared=0%, P=0.765

(c)

Study name	Odds ratio	Lower limit	Upper limit	Z-Value	p-Value
Saremi et al. 2021	0.698	0.325	1.499	-0.922	0.356
He et al. 2020	1.593	0.792	3.202	1.307	0.191
Cury et al. 2009	1.733	0.301	9.982	0.616	0.538
Cury et al. 2007	1.125	0.065	19.496	0.081	0.935
	1.135	0.697	1.848	0.510	0.610

Statistics for each study

Odds ratio and 95% CI

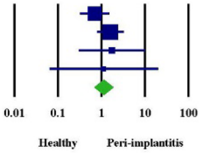

Fixed-effect model, Heterogeneity: Tau-squared=0, I-squared=0%, P=0.443

(d)

Study name	Odds ratio	Lower limit	Upper limit	Z-Value	p-Value
Saremi et al. 2021	0.684	0.360	1.301	-1.157	0.247
He et al. 2020	1.487	0.960	2.303	1.776	0.076
Cury et al. 2009	1.403	0.636	3.095	0.839	0.402
	1.201	0.864	1.669	1.092	0.275

Statistics for each study

Odds ratio and 95% CI

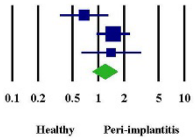

Fixed-effect model, Heterogeneity: Tau-squared=0.09, I-squared=50.07%, P=0.135
